# Gas-Sensitive Properties of ZnO/Ti_2_CT_x_ Nanocomposites

**DOI:** 10.3390/mi14040725

**Published:** 2023-03-24

**Authors:** Elizaveta P. Simonenko, Ilya A. Nagornov, Artem S. Mokrushin, Aleksey A. Averin, Yulia M. Gorban, Tatiana L. Simonenko, Nikolay P. Simonenko, Nikolay T. Kuznetsov

**Affiliations:** 1 Kurnakov Institute of General and Inorganic Chemistry, Russian Academy of Sciences, Moscow 119991, Russia; 2 Frumkin Institute of Physical Chemistry and Electrochemistry, Russian Academy of Sciences, Moscow 199071, Russia; 3 Mendeleev University of Chemical Technology of Russia, Moscow 125047, Russia

**Keywords:** MOS sensor, MXene, ZnO, chemoresistive gas sensors, nanocomposite, selectivity

## Abstract

At present, a new class of 2D nanomaterials, MXenes, is of great scientific and applied interest, and their application prospects are very broad, including as effective doping components for receptor materials of MOS sensors. In this work we have studied the influence on the gas-sensitive properties of nanocrystalline zinc oxide synthesized by atmospheric pressure solvothermal synthesis, with the addition of 1–5% of multilayer two-dimensional titanium carbide Ti_2_CT_x_, obtained by etching Ti_2_AlC with NaF solution in hydrochloric acid. It was found that all the obtained materials have high sensitivity and selectivity with respect to 4–20 ppm NO_2_ at a detection temperature of 200 °C. It is shown that the selectivity towards this compound is best for the sample containing the highest amount of Ti_2_CT_x_ dopant. It has been found that as the MXene content increases, there is an increase in nitrogen dioxide (4 ppm) from 1.6 (ZnO) to 20.5 (ZnO-5 mol% Ti_2_CT_x_). reactions which the responses to nitrogen dioxide increase. This may be due to the increase in the specific surface area of the receptor layers, the presence of MXene surface functional groups, as well as the formation of the Schottky barrier at the interface between the phases of the components.

## 1. Introduction

The development of synthesis methods and research into receptor materials for chemical gas sensors remains one of the most important tasks in the field of functional materials creation [1], because with the development of industry, environmental problems and their impact on human health are becoming more acute. The detection of both toxic gaseous contaminants in the air and marker gases for various serious diseases requires further increases in the sensitivity of sensor materials as the concentrations to be detected move from the ppm level to the ppb and ppt range [2]. For example, ammonia has been found to be the most important respiratory metabolite with a concentration of 833 ppb, followed by acetone (477 ppb), methanol (461 ppb), ethanol (112 ppb), isoprene (106 ppb), acetaldehyde (22 ppb), propanol (18 ppb), etc.

Perhaps an even more urgent task is to create materials with maximum selectivity with respect to a particular gaseous component, since real measurements for environmental or human health monitoring are inevitably associated with operation in complex gaseous media containing many interfering impurities in addition to the target component. Certainly, the use of multi-sensor devices with mathematical processing of a signal by methods PCA (principal component analysis), LDA (linear discriminant analysis) and others, as shown by the studies carried out [3,4,5,6,7], allows researchers to distinguish and define the concentration even when close to structural substances (for example, from a set of aliphatic alcohols) [8,9]. However, the use of receptor materials with the most varied selectivity in the set will significantly increase the accuracy of determination of individual components of the gaseous medium.

Zinc oxide is one of the most popular metal oxide semiconductor sensor (MOS sensor) materials, probably second only to the traditional tin oxide SnO_2_ [10]. It is known to be highly sensitive to toxic analytes such as NO_2_ [11,12,13], H_2_S [14,15], NH_3_ [16,17], as well as to volatile organic compounds [18,19,20,21]. The negative characteristics of this promising substance include rather low selectivity as well as a relatively high detection temperature, mainly in the temperature range of 200 to 400 °C. Studies by many scientific groups show that these disadvantages of ZnO can be compensated to some extent by doping it with various components: n- and p-type semiconductors [22,23,24,25]; noble metal nanoparticles [26,27,28,29,30]; carbon nanotubes or graphene [31,32,33,34,35]; etc.

In recent years, a new class of substances with interesting gas-sensitive properties—the MXenes—has attracted much attention [36,37,38,39,40,41,42,43,44,45,46,47]. These compounds have the general formula M_n+1_C_n_T_x_ (where M is a transition metal from the series Ti, V, Nb, Mo, etc., and T; surface functional groups, most often -F, -OH, =O, -Cl), which can exhibit both metallic and semiconducting properties depending on the method of synthesis [48]. They have been found to exhibit gas sensitivity to some gaseous components (NH_3_, NO_2_, volatile organic compounds or VOC) even at room temperature [49,50,51,52,53], which is extremely useful for the fabrication of sensors on flexible polymer substrates for their incorporation into portable electronic devices [54]. However, individual MXenes are characterized by very low response values (fractions and units of per cent) and sensor response and recovery times (tens of minutes) that are too long for practically important tasks [55,56,57].

Nevertheless, the use of MXenes as a doping component in MOS sensors often allows for the improvement of properties, e.g., in increasing the sensitivity to certain gases or in reducing the operating temperature of the sensor. For example, in [58] it was shown that growing ZnO nanorods on zinc oxide particles deposited on the surface of Ti_3_C_2_T_x_ (content ~36.5%) produces a composite material with high sensitivity to NO_2_ (up to the ppt level) when activated by UV irradiation. The work by of Liu et al. [59] investigated the effect of doping ZnO with low concentrations of Ti_3_C_2_T_x_ (0.5–3%) on its sensitivity to NO_2_. It was found that this leads to the possibility of lowering the detection temperature from 220 to 160 °C—which the authors attribute to an increase in the specific surface area and the number of adsorptions centers—an increase in the electrical conductivity of Ti_3_C_2_T_x_ (which allows rapid transport of charge carriers) and the formation of the Schottky barrier at the interface. In a study by Zhu [60] for ZnO/Ti_3_C_2_T_x_ nanocomposites (the amount of MXene varied from 1 to 3 wt.%), a higher sensitivity to acetone at the operating temperature of 320 °C was observed for samples containing delaminated MXene compared to the accordion-like ones. In the article by Bu et al. [61] it was shown that doping zinc oxide with ~5.7 wt.% Ti_3_C_2_T_x_ does not allow lowering the detection temperature with maximum accuracy below 300 °C. However, a sharp increase of the response value at 100 ppm ethanol compared to single ZnO and Ti_3_C_2_T_x_, low response/recovery times and good long-term stability of the detector were observed.

Improvements in the sensor properties of MXenes have also been observed when they are doped with ZnO nanoparticles [62,63], in particular increased sensitivity to the analyte and reduced response and recovery time of the sensor (especially under UV exposure).

Thus, an analysis of the literature shows that by doping ZnO with MXenes it is possible to improve its gas sensitive properties. However, the few available articles in this field focus on the effect of the most common Ti_3_C_2_T_x_ MXene, which was the first to be synthesized [64], while information on the doping of semiconductor nanodispersed oxides with another two-dimensional titanium carbide Ti_2_CT_x_ is lacking.

The aim of this work is to develop methods for the synthesis of ZnO/Ti_2_CT_x_ nanocomposites containing 1–5 mol% of multilayer MXene and to investigate their gas sensitive properties.

## 2. Materials and Methods

### 2.1. Synthesis and Application

Reagents: powders of metallic titanium (99.9%, 0.5–100 µm, Moscow, Russia, Ruskhim), aluminum (99.2%, 30 µm, Moscow, Russia, Ruskhim), graphite (MPG-8 grade, MPG-8, Technocarb, Chelyabinsk, Russia), potassium bromide KBr (99%, Moscow, Russia, Ruskhim), sodium fluoride NaF (99.9%, Moscow, Russia, Reackhim), hydrochloric acid HCl (>99%, Moscow, Russia, Sigma Tech).

The synthesis of the initial Ti_2_AlC MAX-phase is described in detail in our previous work [65]. In the present work: the powder ratio of *n*(Ti):*n*(Al):*n*(C) = 2:1.2:0.8; the weight of KBr powder added was equal to the total weight of titanium, aluminum and graphite powders; and the synthesis temperature in the molten salt was 1000 °C, the duration was 5 h.

The Ti_2_CT_x_ MXene multilayer was obtained by the method described in [37,40] (without the delamination step). For this purpose, the selective etching of the Ti_2_AlC MAX-phase aluminum layers was carried out by exposure to NaF solution in hydrochloric acid (6M) at 40 °C 24 h. After extraction of the MXenes by centrifugation and washing with distilled water to pH~6–7, their dispersion in ethanol was subjected to an ultrasonic bath for 30 min. Then the powder was dried under a vacuum at 150 °C.

The synthesis of ZnO/Ti_2_CT_x_ composites containing 1, 3 and 5 mol% of MXene (hereafter referred to as samples Z, Z1T, Z3T and Z5T, respectively) was carried out according to the adapted method [66,67]. For this purpose, a sample of MXene sample was dispersed under the influence of ultrasound in a solution of the precursor, monohydrate zinc acetylacetonate monohydrate in butanol, under ultrasonic exposure for 15 min. The reaction system was then heated in an oil bath at 145 ± 5 °C in a round-bottomed flask with a reflux condenser. The duration of solvothermal treatment was 6 h. The resulting precipitate was separated from the mother liquor by centrifugation (3500 rpm, 30 min), washed repeatedly with distilled water and ethyl alcohol solution (95 vol.%) to remove impurity organic fragments, and dried to constant weight under a vacuum at 150 °C.

The resulting ZnO/Ti_2_CT_x_ composite powders were used to prepare the pastes required to form the gas sensitive layers of the sensor. For this purpose, the powder sample was ground in an agate mortar in 1-butanol for 1 h.

Receptor layers were deposited on specialized sensor substrates (Al_2_O_3_ wafers with platinum interdigital electrodes and a platinum heater on the backside) by microplotter printing [36,68,69,70]. The sensors were dried under reduced pressure at a temperature of 150 °C (1 h).

### 2.2. Instrumentation

The following instruments and methods were used to study the physico-chemical properties of the samples obtained. The following equipment was used to study the chemical composition of the surface of the powders and films, and to take microphotographs of the surface of the samples: NVision 40 scanning electron microscope, Carl Zeiss, (Oberkochen, Germany, secondary electron detector, accelerating voltage 1–10 kV); transmission electron microscope (JEOL, JEM-1011, Akishima, Japan); and INCA X-MAX 80 energy dispersive X-ray (EDX) spectrometer, Oxford Instruments (Oxford, UK), accelerating voltage 20 kV. Phase composition was investigated using a D8 Advance (Bruker, Billerica, MA, USA, CuK_α_ = 1.5418 Å, Ni filter, E = 40 keV, I = 40 mA; 2θ range: 5–45°; Resolution: 0.02°; point accumulation time: 0.3 s). The thermal behavior of the synthesized Ti_2_CT_x_-MXene powder was studied using a combined DSC/DTA/TG analyzer SDT-Q600 (TA Instruments) in Al_2_O_3_ crucibles in air and Ar streams (250 mL/min); heating rate 10°/min, temperature range 25–1000 °C.

Raman spectra were obtained using an inVia Reflex «Renishaw» spectrometer (New Mills, Wotton-under-Edge, Gloucestershire, United Kingdom, GL12 8JR) with a 405 nm diode laser as the excitation source. All spectra were recorded in the 100–1750 cm^−1^ range with a spectral resolution of ~3 cm^−1^ through a 50x (NA 0.5, FN 26.5) magnification lens with an irradiated spot diameter of ~2 μm. The incident power was less than 0.2 mW. Lattice: 2400, signal accumulation time was 300 s.

The gas sensitive properties were measured using a specialised precision apparatus. The gas environment in the quartz cell was created using four Bronkhorst gas flow controllers with maximum flow rates of 1, 50, 100 and 200 mL/min. The temperature of the sensor element was controlled by a built-in platinum microheater, which was pre-calibrated using a Testo 868 thermal imager. The resulting film was tested for sensitivity to the following analyte gases: CO, H_2_, CH_4_, NH_3_, benzene, acetone, ethanol and NO_2_. The corresponding test gas mixtures in air were used as the source of the analyzed gases and synthetic air was used to establish a baseline. To measure the signal at different relative humidity (RH), we used a special unit with a barbometer, while the RH of the gas mixture was controlled by a digital flow hygrometer “Excis” (EXIS, Russia, Moscow). The electrical resistance of the oxide films was measured using a Fluke 8846A (6.5-digit precision multimeter) with an upper limit of 1 GΩ. The sensitivity to CO, H_2_, CH_4_, NH_3_, benzene, acetone and ethanol was calculated using the following formula.

S = R_Air_/R, (1)

where R_Air_ is the resistance of the oxide film in the synthetic air medium; R—in the medium with a given concentration of the analyte gas. The response to NO_2_ was calculated using the inverse relationship (1).

The selectivity coefficient was calculated according to the formula:

Sel = S_NO_2__/S_g_, (2)

where S_NO_2__ is the response at 4 ppm NO_2_; S_g_ is the response at a given gas concentration where the highest response value was observed after NO_2_.

## 3. Results and Discussion

### 3.1. Synthesis and Investigation of Ti_2_CT_x_ MXene Powder and ZnO/Ti_2_CT_x_ Composite Powders

**Figure 1:**
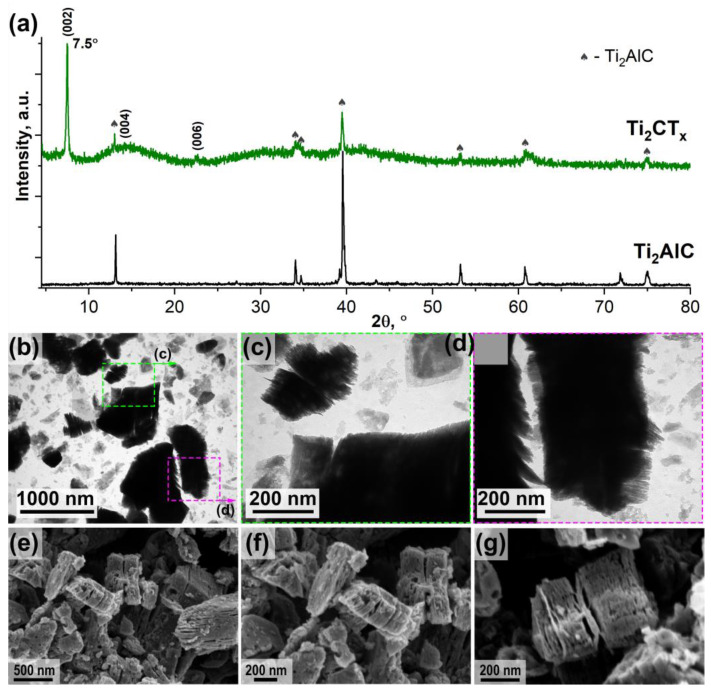
The X-ray powder patterns of the original Ti_2_AlC MAX-phase and the obtained Ti_2_CT_x_ MXene (**a**) and the Ti_2_CT_x_ MXene microstructure particles according to TEM (**b**–**d**) and SEM (**e**–**g**).

As can be seen in Figure 1a, the Ti_2_CT_x_ MXene powder obtained by selective MAX-phase etching contains a small admixture of the parent compound Ti_2_AlC, but there are no common crystalline impurities of Al_2_O_3_, TiC, TiO_2_ and low-soluble aluminum fluorides including Na_5_Al_3_F_14_. The shift of reflex position (002) from 12.9 to 7.5° indicates an increase in the interlayer distance with removal of aluminum layers and formation of surface functional groups.

The investigation of the particle microstructure by TEM (Figure 1b–d) showed that in addition to the multi-layered, coarse, accordion-like Ti_2_CT_x_ particles in the powder, a certain number of low-layered particles with increased defects are present. In addition to MXene layer stacks with relatively long planes (up to 1–1.5 μm), aggregates with much smaller diameters (up to 100–200 nm) are found in approximately equal amounts. A similar conclusion can be drawn from the analysis of the SEM micrographs (Figure 1e–g): the sample contains MXene stacks with different diameters. The small Ti_2_CT_x_ particles could not be fixed by this method. The EDX analysis of the Ti_2_CT_x_ powder shows that the aluminum content does not exceed 1.3 at. % and the ratio *n*(F):*n*(Cl) = 3.7.

**Figure 2:**
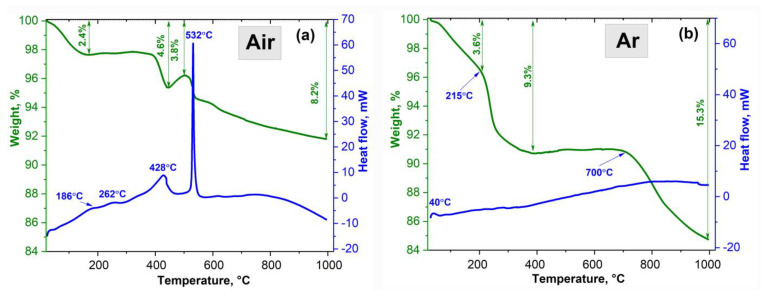
DSC (blue) and TGA (green) curves of Ti_2_CT_x_ MXene obtained by thermal analysis in air flow (**a**) and argon (**b**).

The thermal behavior of Ti_2_CT_x_ multilayer powder has been studied in a flow of inert gas and air, shown in Figure 2. As can be seen, in both cases a loss of mass begins at low temperatures, which is probably related primarily to the desorption of water molecules. In this case, up to the temperature range of 150 to 170 °C, the TGA curves for air and argon coincided, while at higher temperatures the mass of the sample in the air flow was significantly higher than in the argon flow. This may be related to the onset of oxidation of the most chemically active particles of the MXene low-layer MXene present in the sample. At temperatures > 385 °C during heating in the air flow (Figure 2a), the stabilization of the mass of the sample in the air flow is followed by a decrease in mass, then an increase (>445 °C) and again a decrease (>505 °C). These mass changes are associated with the parallel processes of detachment of MXene surface functional groups (mass decrease) and oxidation of two-dimensional titanium carbide, accompanied by exothermic effects with a maximum at temperatures of 428 and 532 °C. The total cumulative mass loss at 1000 °C was 8.2%.

However, when heated in an argon stream (Figure 2b), a stepwise decrease in mass is observed, which at low temperatures (<200–220 °C) is probably mainly due to the removal of adsorbed water molecules. Heating to a higher temperature resulted in an increase in the rate of mass decrease due to the removal of surface hydroxyl groups, which is completed at 380 °C. A further step in the mass loss is observed at temperatures > 700 °C, probably due to the decomposition of the MXene by detachment removal of the fluoride and chloride functional groups already present.

**Figure 3:**
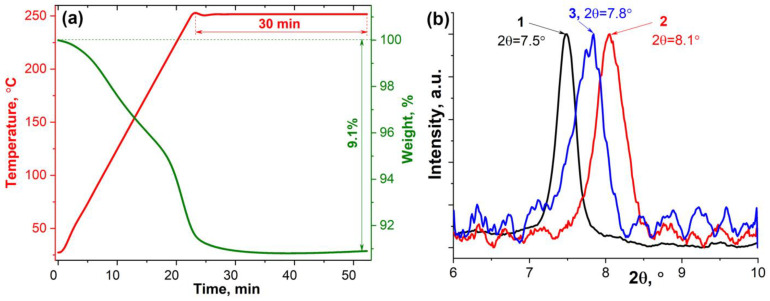
TGA curve after heating the resulting accordion-like Ti_2_CT_x_ MXene powder in an argon flow to the temperature 250 °C and holding for 30 min (**a**), and also sections of the X-ray diagram in the interval 2θ = 6–10° showing the reflex position (002) (**b**) in the initial powder (1), after heating in an argon flow to 250 °C for 30 min (2) and after holding the sample in a humid atmosphere for 2 days (3).

In order to study the behavior of Ti_2_CT_x_ powder when heated to relatively low temperatures, at which water desorption and detachment of surface OH-groups are assumed, we studied the change in MXene reflex position (002) after its incubation in an argon stream at 250 °C under DSC/TGA experiment (heating rate was 5°/min), shown in Figure 3. It is shown that the mass loss in this regime was 9.1% (Figure 3a), which is practically the same as that observed for thermal analysis in an argon stream above a temperature of 380 °C (Figure 2b). At the same time, the mass of the sample was already stabilized after 15 min of heating to the temperature 250 °C. X-ray diffraction of the obtained sample in the interval 2θ = 6–10° confirmed the shift of the reflex position (002) of the Ti_2_CT_x_ MXene from 7.5° to 8.1°, which indicates the decrease of the interlayer distance from 11.8 to 11.0 Å as a result of the desorption of water molecules. At the same time, keeping the sample in a humid atmosphere for 2 days leads to the initiation of the opposite process, shifting the reflex (002) position towards lower angles from 8.1 to 7.8°. The data obtained correspond to the situation described in [71] for another two-dimensional titanium carbide, Ti_3_C_2_T_x_, the shift resulting from the purging of the chamber with nitrogen for 200 min to remove adsorbed and intercalated molecules of reflex position (002) and from the corresponding reduction of the interlayer distance by 0.84 Å.

**Figure 4:**
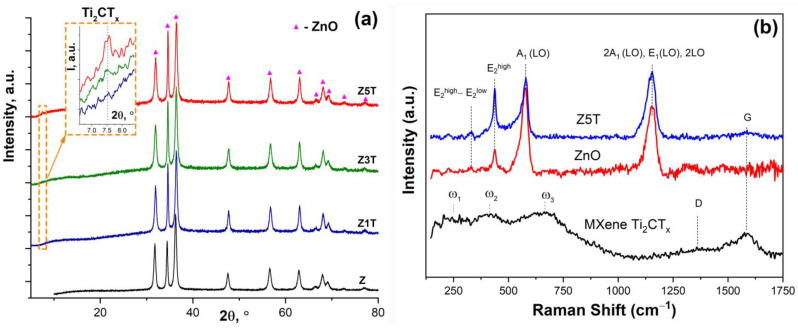
X-ray diffraction patterns of the obtained powders Z, Z1T, Z3T and Z5T (**a**) and Raman spectra of Ti_2_CT_x_ MXene powders and Z and Z5T samples (**b**).

The phase composition of the obtained ZnO/Ti_2_CT_x_ composite powders was investigated by XRD (Figure 4a) and Raman spectroscopy (Figure 4b). The X-ray diffraction patterns of all samples show intense but broadened reflexes of well-crystallized zinc oxide with wurtzite structure (Figure 4a), whereas for the Z5T sample (containing maximum amount of Ti_2_CT_x_–5 mol.%) at 2θ = 7.5° an additional low intensity reflex of accordion-like Ti_2_CT_x_ is observed. All the Raman spectra of the powders obtained were recorded at low power to avoid oxidation of the oxides by the laser, so the spectra were obtained with some noise. As can be seen from Figure 4b, the spectra of the ZnO and Z5T samples show a characteristic set of ZnO peaks of the wurtzite phase, at 333, 438, 578 and 1154 cm^−1^ belonging to modes (E_2_^high^ -E_2_^low^), E_2_^high^, A_1_ (LO) and [2A_1_ (LO), E_1_ (LO), 2LO], respectively [72,73]. The Raman spectrum of the Ti_2_CT_x_ MXene powder ω_1-3_ with broad MXene own bands with maxima at 247, 416 and 668 cm^−1^ [74,75], and a weakly intense D- and intense G-band at 1361 and 1583 cm^−1^ [76], related to carbon modes, are present. The D- and G-bands are characteristic for MXenes and other carbon systems with sp^2^-hybridization of the C-C bond. The spectrum of the Z5T sample lacks the intense ω_1-3_ and D-bands of MXenes due to the low content of MXene, but the G-band is clearly visible, which is a sign of the presence of carbon structures in the nanocomposites (in this case, MXenes).

**Figure 5:**
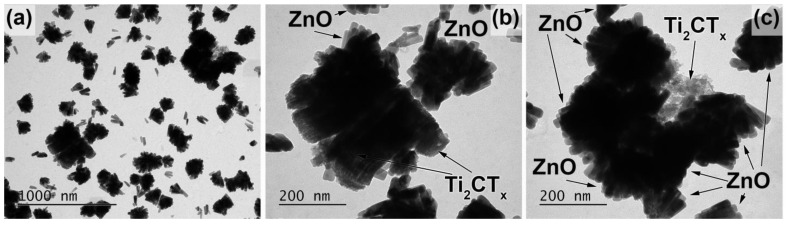
Particle morphology of ZnO/Ti_2_CT_x_ (Z5T) composite powder by TEM.

TEM of powder sample Z5T with maximum MXene content (Figure 5) shows that in addition to the rod aggregates (diameter 10–30 nm and length 40–90 nm) common to ZnO nanoparticles synthesized by the above method, the sample also contains buds [77] in the sample in which these ZnO aggregates are attached to accordion-like (Figure 5b) and small layered MXene particles (Figure 5c). Despite the low MXene dopant content in the compositions, such aggregates in which there are phase contacts between ZnO and Ti_2_CT_x_ occur quite frequently in the total particle mass, which may be due to the fact that Ti_2_CT_x_ acted as a seed for the formation of the ZnO phase.

**Figure 6:**
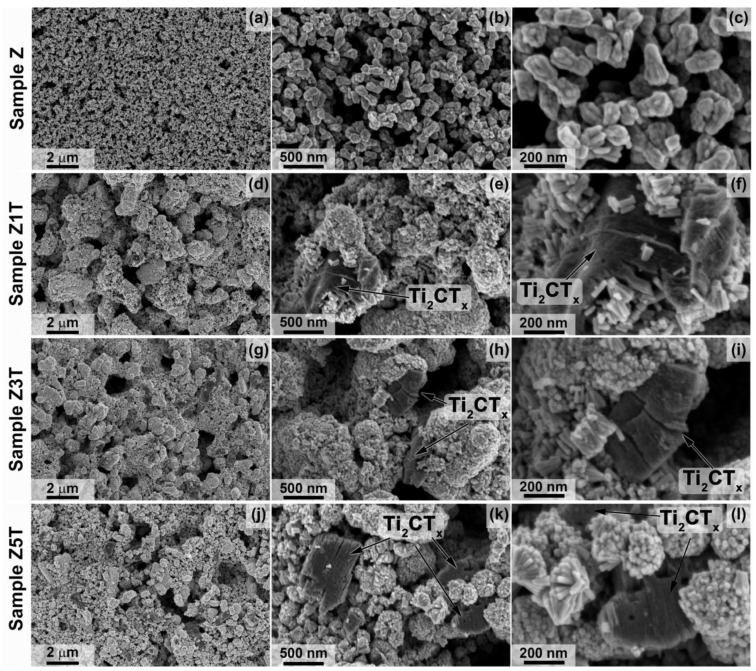
Microstructure of the receptor layers of samples Z (**a**–**c**), Z1T (**d**–**f**), Z3T (**g**–**i**) and Z5T (**j**–**l**) deposited on a sensor substrate by microplotter printing, as determined by SEM.

**Figure 7:**
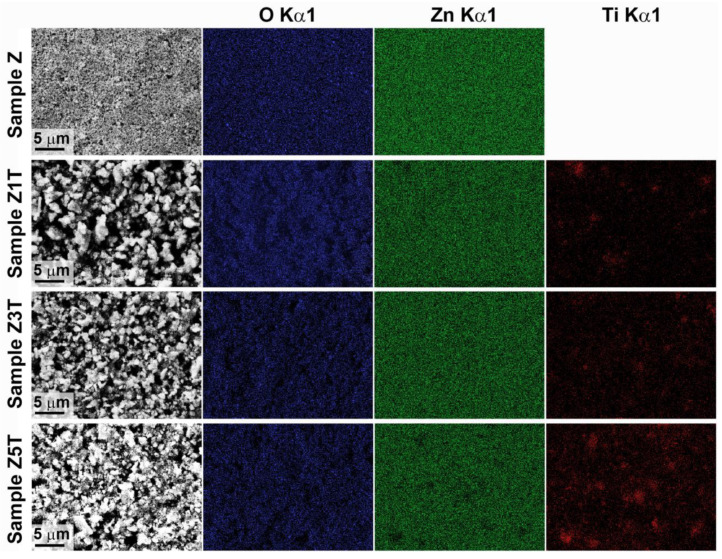
Distribution of elements O, Zn and Ti obtained by EDX mapping for coating samples Z, Z1T, Z3T and Z5T.

Scanning electron microscopy was performed on additively deposited receptor layers of ZnO and ZnO/Ti_2_CT_x_ compositions on the surface of specialized substrates (Figure 6). As can be seen from the micrographs, when doped with MXene, uniform ZnO aggregates in the form of a porous and uniform layer (Figure 6a–c) are replaced by a less regular aggregate size distribution (Figure 6d–l). At the same time, the size and shape of the aggregates forming the receptor layer is determined by the MXene stacks morphology (length 1–2.5 μm and thickness 0.8–1.8 μm, which corresponds to the size of the initial Ti_2_CT_x_ aggregates including the ZnO ‘coat’, Figure 1), whose packing leads to an increase in the average pore size. Another peculiarity of Z1T-Z5T samples is that due to the less dense packing of the ZnO rods in the cross-links, the proportion of unaggregated particles increases. As the ZnO content increases, the number of accordion-like ZnO particles appearing on the surface of the composite layer (partially uncoated by ZnO rods) increases. The size of the zinc oxide rods themselves does not vary significantly with changes in the composition of the *n*(ZnO):*n*(Ti_2_CT_x_) ratio: the length of a single rod varies from 50 to 100 nm and the thickness varies from 10 to 30 nm. The mutual distribution of the elements Zn, Ti and O is shown in Figure 7. As can be seen, for the sample Z5T with the highest Ti_2_CT_x_ content the following is observed: the absence of ZnO is clearly visible in the place of titanium-containing inclusions, i.e., zinc oxide nanoparticles are localized around rather large accordion-like MXene aggregates.

### 3.2. Chemoresistance Properties of ZnO/Ti_2_CT_x_ Composites

The obtained films of ZnO/Ti_2_CT_x_ composition showed electrical conductivity, which allowed the study of their chemoresistive gas-sensitive properties only at elevated temperatures. The main phase of the studied nanocomposites is ZnO, a broad band n-type semiconductor (E_g_ = 3.37 eV), therefore the electrophysical properties of ZnO/Ti_2_CT_x_ nanocomposites are mainly determined by ZnO, which is characterized by medium and high detection operating temperatures. Modification of ZnO with Ti_2_CT_x_ MXene, which has metallic conductivity, did not significantly reduce the electrical resistance. All other chemosensory measurements were carried out at the optimum operating temperature of 200 °C, at which sufficient nanomaterial resistance was observed for the measurements and the structure of the Ti_2_CT_x_ MXene was preserved (oxidation to TiO_2_ starts at temperatures around 300 °C to TiO_2_ [40]).

**Figure 8:**
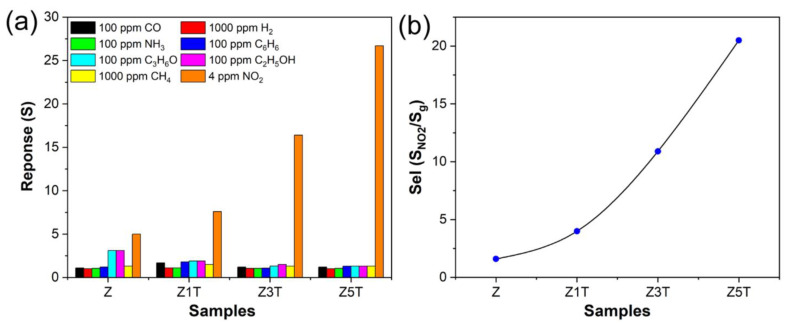
Selectivity histogram constructed from the responses to different gases (H_2_, CH_4_, CO, C_6_H_6_, NH_3_, ethanol, acetone and NO_2_) (**a**) and the dependence of the selectivity coefficient (Sel) of the receptor layers of samples Z, Z1T, Z3T and Z5T (**b**); detection temperature 200 °C.

In a first step, detection responses were obtained for all samples for a broad group of analyte gases at specified concentrations (1000 ppm: H_2_ and CH_4_; 100 ppm: CO, C_6_H_6_, NH_3_, ethanol, acetone and 4 ppm NO_2_). The highest response, which was significantly higher than for all other gases, was obtained for NO_2_. Figure 8a shows a histogram of the selectivity, which indicates that the individual zinc oxide also has a high sensitivity with respect to other gases besides NO_2_ (the highest response was obtained for 100 ppm ethanol and acetone S = 3.1, while the response for 4 ppm NO_2_ was 5.0). When ZnO is modified with MXene, the response to 4 ppm NO_2_ increases to 7.6, 16.4 and 26.7 for Z1T, Z3T and Z5T respectively, and the response to other gases decreases to 1.3–1.9. For a convenient numerical estimation of the selectivity, a special coefficient (Sel) was calculated according to Formula (2); the higher the coefficient, the greater the difference between the responses to different gases (in this case, the responses to other gases were compared with the response to 4 ppm NO_2_) and the better the selectivity. Figure 8b shows the dependence of Sel on the MXene content of ZnO/Ti_2_CT_x_ nanocomposites. It was found that the selectivity to NO_2_ detection improves with increasing MXene content in the nanocomposites.

**Figure 9:**
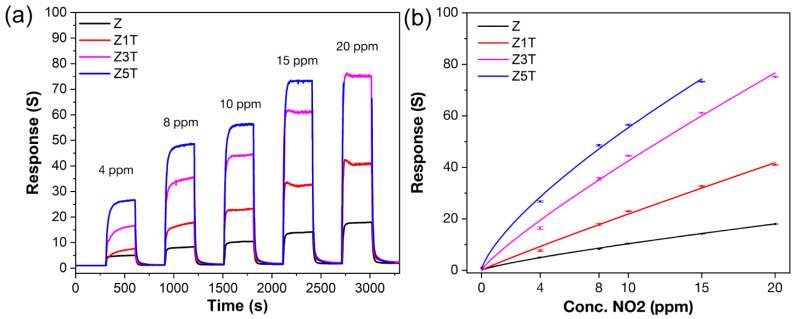
Sensitivity to 4–20 ppm NO_2_ (**a**) and response as a function of concentration (**b**) of ZnO/Ti_2_CT_x_ coatings at 200 °C detection temperature.

Figure 9a shows the experimental data on the sensitivity of the obtained receptor materials to different concentrations of NO_2_. As can be seen, the zinc oxide-based coatings exhibit a high response over a wide range of very low NO_2_ concentrations (4–20 ppm), exceeding the threshold limit value (TLV) recommended by the National Institute for Occupational Safety and Health [78]. It was found that with increasing concentrations from 4 to 15 ppm NO_2_, the response value increased from 5 to 14.2, from 7.6 to 32.7, from 16.4 to 61.2 and from 26.7 to 73.3 for samples Z, Z1T, Z3T and Z5T respectively. Thus, the response to NO_2_ increased significantly over the whole concentration range with increasing as the MXene content was increased. For sample Z5T, which showed the highest sensitivity to NO_2_, the response cut off at 20 ppm NO_2_ because the electrical resistance of the material at 20 ppm NO_2_ exceeded 1 GΩ, which did not allow the gas sensitivity of the material to be correctly estimated on the equipment used in this study.

Figure 9b shows the dependence of the response on the concentration of NO_2_ in the gas mixture. The observed dependence of the sensor response on the NO_2_ concentration is well described by the Freundlich isotherm equation: S = kC^a^, where *k* and *a* are proportional and exponential constants representing the adsorption capacity and the adsorption enhancement, respectively. The character of the obtained dependences agrees well with the data previously obtained for ZnO-based nanocomposites [11,18].

Analyzing the shape of the received signals, it can be seen that at 4–8 ppm NO_2_ the shape of the signals from all samples is bell-shaped, whereas at 10–20 ppm it is almost rectangular. The shape of the signals has a direct effect on the response time (t_90_). For example, when detecting 4–8 ppm NO_2_, the ZnO sample shows a t_90_ in the range of 40–45 s and at 10–20 ppm it is about 30–35 s. When modified with Ti_2_CT_x_ MXene at low NO_2_ concentrations (4–8 ppm), the response times increase to 93–172, 55–124 and 45–68 s for Z1T, Z3T and Z5T respectively. When 10–20 ppm NO_2_ is detected, the response time decreases significantly to ~22, 25 and 35 s, respectively. Thus, when ZnO is modified with Ti_2_CT_x_ MXene, there is an increase in response time (compared to single ZnO) when detecting low concentrations and a decrease when detecting higher concentrations.

Since zinc oxide is the main gas sensitive material in ZnO/Ti_2_CT_x_ nanocomposites, the detection mechanism will be determined to a greater extent by the behaviour of ZnO. To describe the NO_2_ detection mechanism, the generally accepted model related to the presence of ion-adsorbed oxygen (O^−^) and electron depletion layer (EDL) on the ZnO surface (*n*-type semiconductor) should be used [79]. Sensitivity of single ZnO high response in the detection to NO_2_ by single ZnO is related to the reactions that take place on the surface of the material. Of all the metal oxide materials used as receptors in MOS sensors, ZnO probably has the strongest basic properties [80]. In the work of Mei Chen et al. [81] and Liu et al. [82], as in our previous study [18], it was shown that the highest response to NO_2_ is observed in the operating temperature range of 125–200 °C. Due to the strong basic properties of ZnO, the NO_2_ influx leads to the formation of nitrite (NO_2_^−^), and then nitrate (NO_3_^−^) in the first few minutes, involving various defects in the ZnO crystal lattice and ion-adsorbed oxygen. The formation of nitro-groups occurs with the participation of electrons from the conduction band of ZnO, which explains the high chemoresistance response. The modification of zinc oxide by the accordion-like Ti_2_CT_x_ MXene leads to a consistent increase in sensitivity to NO_2_, which can be associated primarily with a marked increase in the specific surface area of the receptor material, leading to an increase in the number of adsorption centers and the diffusion rate of gases in the volume of the receptor layer. In addition, the presence of polar functional groups in the MXene structure can further contribute to the adsorption of gases capable of forming hydrogen bonds. The formation of heterojunctions at the interface between the components should lead to charge separation and increase the sensitivity to electron donor or acceptor gases [59,62,63,82,83,84].

Table 1 compares the gas sensing characteristics of ZnO/Ti_2_CT_x_ and ZnO/Ti_3_C_2_T_x_ nanocomposites presented in the literature. As can be seen, ZnO/Ti_3_C_2_T_x_ nanocomposites are the main ones described in the literature. It was found that the vast majority of materials exhibit an increased response to NO_2_, which is also typical of individual zinc oxide. Nevertheless, the response to NO_2_ of ZnO/Ti_2_CT_x_ nanocomposites obtained in this study is significantly higher than that of their analogues. The Sel parameter, which characterizes selectivity towards this gaseous analyte, was also estimated to be higher for ZnO/Ti_2_CT_x_ nanocomposites obtained using the solvothermal method as compared to the analogues described in the literature.

## 4. Conclusions

Using atmospheric pressure solvothermal synthesis, composite powders in which nanodispersed zinc oxide was doped with 1–5 mol% of the accordion-like Ti_2_CT_x_ MXene were synthesized, and their phase composition and microstructure were studied. It was found that the MXene powder addition in the synthesis process resulted in the formation of ZnO aggregates with a more porous structure compared to individual ZnO. This is probably due to the fact that the growth of ZnO particle nucleation on the porous MXene surface limited the consolidation.

The study of the chemoresistive properties of the obtained samples at the detection temperature of 200 °C for the most practically required analyte gases (explosive, toxic inorganic compounds and VOCs) revealed an extremely high sensitivity of all materials to NO_2_. At the same time, the selectivity increased with the increase of the MXene component: the ratio of the response value at 4 ppm NO_2_ to the second highest response value (at 100 ppm acetone/ethanol) increased from 1.6 (ZnO) to 20.5 (sample Z5T). The response time for the detection of 4–20 ppm NO_2_ for single ZnO was 30–45 s. When ZnO is modified with Ti_2_CT_x_ MXene, there is an increase in the response time for the detection of low concentrations (up to 45–124 s, 4–8 ppm NO_2_) and a decrease for the detection of higher concentrations (up to 22–35 s). In addition to the high selectivity with increasing MXene content in the composite powder, the sensitivity to NO_2_ increased over the whole range of concentrations studied (4–20 ppm). For example, the response value increased by 4 ppm from 5 for ZnO to 26.7 for the sample containing 5 mol% Ti_2_CT_x_. This can be attributed to the increase in the specific surface area of the material, which causes an increase in the number of adsorption centers and the rate of analyte diffusion, as well as to the presence of polar functional groups on the MXene surface and the formation of a Schottky barrier at the interface.

## Figures and Tables

**Table 1:** Comparison of gas sensing properties of ZnO/Ti_2_CT_x_ and ZnO/Ti_3_C_2_T_x_ nanocomposites reported in the literature.

Year	Composition	Gas	Conc., ppm	Temp, °C	Response	Response Time (s)	Sel ^1^	Ref.
2021	ZnO—Ti_3_C_2_T_x_	NO_2_	100	RT	41.39 ^2^	34	~8	[62]
2022	ZnO—Ti_3_C_2_T_x_	NO_2_	20	RT	367.63 ^2^	22	~6	[63]
2022	ZnO—36.5% Ti_3_C_2_T_x_	NO_2_	0.05	RT, UV	81 ^2^	17	~4	[58]
2022	ZnO—2% Ti_3_C_2_T_x_	Acetone	100	320	14.4 ^3^	8	~1.03	[60]
2022	ZnO—Ti_3_C_2_T_x_	NO_2_	8	160	3.6 ^3^	191	~3.6	[59]
2023	ZnO—3.7% Ti_3_C_2_T_x_	Ethanol	50	300	59 ^3^	22	~5	[61]
2023	ZnO—(1–5%)Ti_2_CT_x_	NO_2_	20	200	>73.3 ^3^	22	20.5	This work

^1^ Formula used for Sel: S_Ranalyte_/S_R2_, where S_R2_ is the highest response among the gases tested in this article, excluding response to gas analyte. ^2^ Formula used for S_R_ = (ΔR/R_a_) × 100. ^3^ Formula used for S_R_ = R_a_/R_g_.

## Data Availability

Not applicable.
